# Supplementary material for: Development of chemokine network inhibitors using combinatorial saturation mutagenesis
Source: Commun Biol. 2025 Apr 3;8:549. doi: 10.1038/s42003-025-07778-6 (PMC11969024; doi:10.1038/s42003-025-07778-6)
Supplement: Supplementary file 4 — Supplementary Data 2 [file 42003_2025_7778_MOESM4_ESM.zip › SupplementaryData3/scripts/xattune_dr.tar!/./script/analysis/JK/JK_E181_CXCL9_ATC_plot.pdf]

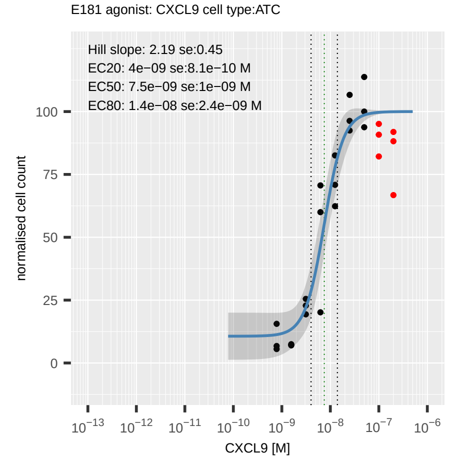

E181 agonist: CXCL9 cell type:ATC
Hill slope: 2.19 se:0.45
EC20: 4e−09 se:8.1e−10 M
EC50: 7.5e−09 se:1e−09 M
EC80: 1.4e−08 se:2.4e−09 M
normalised cell count
100
75
50
25
0
10^{-13} 10^{-12} 10^{-11} 10^{-10} 10^{-9} 10^{-8} 10^{-7} 10^{-6}
CXCL9 [M]
